# Brain Activity During Unilateral Physical and Imagined Isometric Contractions

**DOI:** 10.3389/fnhum.2019.00413

**Published:** 2019-11-26

**Authors:** Jonathan A. Martinez, Matthew W. Wittstein, Stephen F. Folger, Stephen P. Bailey

**Affiliations:** ^1^Department of Exercise Science, Elon University, Elon, NC, United States; ^2^Department of Physical Therapy Education, Elon University, Elon, NC, United States

**Keywords:** imagery, EEG, isometric, prefrontal cortex, sensorimotor cortex

## Abstract

By convention, it is believed that the ipsilateral side of the body is controlled by the contralateral side of the brain. Past studies measuring brain activity primarily recorded changes before and after an intervention is performed on one side of the body within one hemisphere (usually the contralateral) of the brain. The purpose of this investigation was to observe the brain activity within the left and right hemispheres of the prefrontal and sensorimotor cortices during physical and imagined, dominant and non-dominant unilateral isometric elbow flexion. Fifteen right hand dominant individuals (six males and nine females) between the ages of 18 and 21 performed four different isometric contractions of their biceps brachii at a preacher curl bench: dominant physical contraction (DomCon), non-dominant physical contraction (NonCon), dominant imagined contraction (DomImagine), and non-dominant imagined contraction (NonImagine). Each contraction was sustained for 5 s followed by 30 s of rest. Motor activity-related cortical potential (MRCP) and event-related spectral perturbation (ERSP) within the right and left hemispheres of the sensorimotor and prefrontal cortices were determined for each condition at 500–1,000 ms and 2,000–2,500 ms after initiation of contraction. MRCP and ERSP were both changed at the 500–1,000 ms time window for all conditions. Changes in the 2,000–2,500 ms window were most consistently observed during physical contractions. While the changes during DomCon occurred in the left (contralateral) side of the brain, the greatest changes observed in MRCP and ERSP occurred in both sides of the brain during the NonCon condition. Further understanding of bilateral changes in brain activity during unilateral tasks is valuable for improving rehabilitation practices through mental and physical exercise.

## Introduction

It is conventionally believed that the right side of the body is controlled by the left side of the brain and vice versa, giving rise to the belief that the body operates through laterality. However, this may not be entirely true. Functional magnetic resonance imaging (fMRI) has shown blood oxygenation levels increase bilaterally during performance of complex physical unilateral tasks, specifically when performed on the non-dominant side of the body (Van Impe et al., 2009). Additionally, studies observing cross-education—the transfer of strength, mobility, and neural control from physically or mentally training one side of the body to the nontrained side—have theorized that unilateral tasks develop a motor schema in higher centers of the brain. This may result in the improvement of either version of the task experienced on either side of the body (Sidaway and Trzaska, 2005; Lee and Carroll, 2007; Cirer-Sastre et al., 2017). These observations suggest that the brain may possess a similar response when performing a mental or physical version of the task, as well as some degree of bilateral activity for this cross-education effect to occur.

One of the most common methods used to measure brain activity during physical tasks is observing motor activity-related cortical potential (MRCP) *via* electroencephalography (EEG). When looking at MRCP waveforms, their amplitudes characteristically begin with a negative slope prior to voluntary movement. The negative deflection corresponds with the activation of cortical regions for the execution of the desired task. They are then typically followed by an increasing positive deflection representative of sustained maximal muscle contraction (Gilden et al., 1966). Several EEG studies have investigated MRCP magnitudes in the sensorimotor and supplementary motor regions when executing physical contractions with and without implementation of a physical training intervention. Ranganathan et al. (2004) found an increase in MRCP magnitudes within the sensorimotor region after a finger abduction physical training intervention. Likewise, other research using MRCP amplitudes to evaluate brain activity during elbow flexion has identified the sensorimotor cortex and somatosensory areas of the frontal and parietal lobes to be important regions during these tasks (Siemionow et al., 2000; Fang et al., 2001). When EEG MRCP values were cross-referenced with fMRI scans during a thumb flexion and extension task, both readings displayed activity in the supplementary motor area and motor cortex (Yue et al., 2000). Combined, these studies demonstrated that tasks involving voluntary movement will show MRCP activation within the sensorimotor and supplementary motor regions with different magnitudes depending on phase of movement, training for task, and anatomical location of movement.

Understanding how mental practice or training may influence brain activity would elucidate the plausible applications of mental practice in a rehabilitative setting. Yet, only a few studies have monitored MRCP changes in similar brain regions during a physical contraction after completion of a mental training intervention. MRCP magnitudes within the sensorimotor and frontal regions increase after both a finger and elbow mental training intervention (Ranganathan et al., 2004). Yao et al. (2013) also found MRCP magnitudes to increase significantly in elbow flexion from a mental imagery training intervention within the supplementary and contralateral sensorimotor regions. Mental imagery has also been found to stimulate activity in the motor cortex in some cases (Sharma et al., 2006). Internal mental imagery can promote significant changes in the activity of the supplementary and sensorimotor regions of the brain as it requires the recreation of kinesthetic feeling (Yao et al., 2013); however, evoked changes are not as large as those evinced from physical training (Ranganathan et al., 2004). Although unilateral mental training has been shown to impact brain activity during the physical execution of a task, it is unclear what is responsible for the changes in brain activity after completion of the mental training intervention.

Time-frequency analysis, another popular approach for interpreting brain activity in EEG data, describes changes in frequency band activity through the phenomena of event-related de-synchronization (ERD) and event-related synchronization (ERS; Kubitz and Mott, 1996; Jeon et al., 2011). ERD and ERS are commonly associated with the planning and execution phases of the movement, respectively. The frequency bands most commonly associated with these phases are alpha (7.5–12 Hz) and beta (13–30 Hz) bands (Kubitz and Mott, 1996; Jeon et al., 2011). Alpha bands decrease during instances of high arousal and increase during instances of low arousal (Kubitz and Mott, 1996; Neuper and Pfurtscheller, 2001; Chen et al., 2013). Conversely, beta bands associated with physical movement—planning and execution—have been observed to increase within the frontal region of the brain during physical activity (Moraes et al., 2007; Bailey et al., 2008). Mental contractions change alpha and beta bands within the sensorimotor region (Bian et al., 2018).

Several studies have described frequency band changes during mental and physical tasks within the sensorimotor region. Bian et al. (2018) observed ERD in alpha and beta bands in the sensorimotor region during a mental opening–closing task of the right hand. The extent to which ERD was observed is dependent on the type of guidance provided (verbal, video, or audiovisual) and the complexity of the task—more complex tasks (i.e., those requiring more planning) demonstrate more ERD (Bian et al., 2018). Nakayashiki et al. (2014) reported ERD/ERS in beta bands across the sensorimotor region during a physical opening–closing right hand gripping task. Their results indicated increases in ERD/ERS during both slow and fast closing of the hand and when grasping loads increased (Nakayashiki et al., 2014). In comparison, Chen and associates found significant correlations between mental hand rotations and suppressed alpha band activity in the central parietal region of both hemispheres in the brain; however, there was no significant correlation between the mental hand rotation and beta levels (Chen et al., 2013). In regard to physical tasks, Neuper reported observing an increase in beta bands during a voluntary foot lifting and finger flexion task in the somatosensory regions of the brain (Neuper and Pfurtscheller, 2001). Similarly, Wang et al. (2015) observed increased activity in the alpha and beta bands during the sustained portion of contraction compared to initiation. Another study measuring alpha and beta bands during both mental and physical hand clenching tasks on the dominant and non-dominant side found bilateral changes within the sensorimotor region (Yuan et al., 2010). More specifically, alpha band activity during the physical contraction of the non-dominant (left) side presented the greatest change (Yuan et al., 2010). Collectively, these investigations suggest that there are distinct changes in frequency band activity that can be associated with specific phases of both physical and mental tasks.

Investigations describing brain activity during unilateral execution of physical and mental tasks on both the dominant and non-dominant sides of the body are critical in order to fully understand the relationship between the two types of tasks and the extent of laterality on which the brain operates. Although studies have examined unilateral physical and mental tasks *via* EEG, the majority of these investigations only report brain activity prior to and after a unilateral training intervention or simple movement. Few efforts have been made to document changes in brain activity during both mental and physical tasks within the same investigation prior to the application of an intervention. Furthermore, studies often fail to contrast brain activity when executing the task on the dominant vs. non-dominant side of the body. The purpose of this study was to analyze brain activity in the sensorimotor and prefrontal cortices during physical and imagined isometric contractions of the biceps brachii in the dominant and non-dominant limbs *via* MRCP and time-frequency analysis. It was hypothesized that mental and physical contractions would precipitate comparable changes in MRCP waveforms and depression of alpha band activity before the task. It was also hypothesized that similar MRCP amplitudes and frequency bands would be observed during the execution of the physical dominant and non-dominant contractions on their respective contralateral side of the brain. Likewise, it was expected that mental dominant and non-dominant contractions would exhibit a similar pattern of response.

## Materials and Methods

### Participants

A total of 15 young healthy individuals (six males and nine females; age = 18.8 ± 1 years, height = 170.5 ± 11 cm, mass = 71.2 ± 13.1 kg, left grip strength = 29.6 ± 8.7 kg, and right grip strength = 32 ± 9.6 kg) participated in this investigation. Exclusion criteria for participants included engagement in any upper-body weight lifting or rigorous exercise routines within 6 months prior to the study or being ambidextrous. All participants in this study were right hand dominant. Additionally, participants with any neurologically or biomechanically impairing diseases, injuries, or medical prescriptions were not eligible. Prior to enrollment, all participants provided informed consent and answered a health questionnaire concerning demographic and clinical information approved by the Institutional Review Board of Elon University.

### Experimental Procedure

Participants were instructed to sit at a preacher curl bench bearing a preacher bar of 125 lbs. The weight was chosen to ensure participants could not lift the bar with one hand. A computer monitor was placed in front of the participants that provided automatic testing directions throughout the session. Participants sustained four different isometric contractions for 5,000 ms five times each, with 30,000 ms rest in between each contraction in the following order: dominant physical (DomCon), non-dominant physical (NonCon), dominant imagined (DomImagine), and non-dominant imagined (NonImagine). Physical contractions consisted of attempting to lift the preacher bar only with the appropriate hand. Mental contractions consisted of participants resting the specified arm in an extended position atop the arm cushion and imagining performing an isometric contraction with the appropriate hand. Electromyography (Delsys, Inc., Boston, MA, USA) of the biceps brachii and triceps brachii were observed and confirmed that participants demonstrated bursts of muscle activity during physical contractions and minimal activity during imagined contractions (Supplementary Figure S1, Table S1).

### EEG Measurement

**Figure 1:**
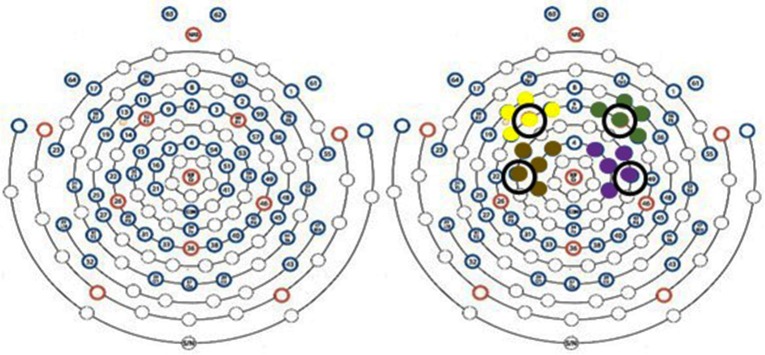
EEG scalp sensor layout. Yellow, green, brown, and purple sensor clusters correspond to left prefrontal [9 (F1), 11 (AF3), **12 (F3)**, 13 (F5), 14 (FC5)] right prefrontal [2 (AF4), 3 (F2), 57 (FC6), 59 (F6), **60 (F4)**], sensorimotor sensors [7 (FC1), 15 (FC3), 16 (C1), **20 (C3)**, 21 (CP1)] and right sensorimotor cortices [41 (CP2), **50 (C4)**, 51 (C2), 53 (FC4), and 54 (FC2)], respectively. Circled electrodes were used for time-frequency analysis.

EEG was collected at 250 Hz using an EGI Geodesic System 300 equipped with a HydroCel Geodesic 64 Channel net and Net Station 5.4 software (Eugene, OR, USA). For this investigation, changes in EEG amplitude were averaged over electrodes in areas associated with the right and left prefrontal and sensorimotor cortices (Figure 1). The specific EGI electrode locations and their corresponding international 10-10 reference as determined by Luu and Ferree (2005) are described in Figure 1. For time-frequency analysis, representative electrodes from each brain region were used for analysis, specifically channel 12 (F3) representing the left prefrontal cortex, channel 60 (F4) representing the right prefrontal cortex, channel 20 (C3) representing the left sensorimotor cortex, and channel 50 (C4) representing the right sensorimotor cortex. Additionally, the references to side of the brain are made using right and left. This approach was taken in an effort to minimize confusion during discussion of responses to dominant and non-dominant contractions. For example, since all participants were right-handed, the left side of the brain during dominant contractions represents the contralateral side while the right side of the brain represents the ipsilateral side.

E-Prime was used to provide instructions in a timed and consistent manner so that accurate epoch time stamps for stimulus conditions were automatically generated. Following collection, all files were bandpass filtered (0.1–50 Hz) and then exported into EEGLab (Swartz Center for Computational Neuroscience, UC San Diego, La Jolla, CA, USA) where independent component analysis (ICA) was performed and bad components (eye blink, eye movement, muscle, heart, and line noise) were removed (Onton and Makeig, 2006).

After ICA, the data were corrected to baseline, artifact was removed, and an average reference was applied. The EEG signal was then analyzed for changes in amplitude and frequency during physical and imagined contractions.

#### Changes in Amplitude

Mean amplitude was calculated for two epochs after the initiation of contraction, between 500–1,000 ms and 2,000–2,500 ms. The epochs used for analysis were determined after visual inspection of grand means for all four conditions. These epochs represent the time points after contraction where the average EEG signal deviated most from baseline. These time frames are also consistent with the time frames described by Mizuguchi and Kanosue (2017) that have previously been associated with motor imagery.

#### Changes in Frequency

Time-frequency analysis was performed using the *newtimef* function (3-cycle Hanning-tapered window wavelets in 0.5-s sets) from the EEGLab toolbox and event-related spectral perturbation (ERSP) was calculated (Delorme and Makeig, 2004). Spectral power was averaged across trials and converted to log power (dB). ERD/ERS was assessed with respect to a −200 to 0 ms pre-stimulus period. Power spectral density (PSD) was compared across the conditions in the alpha (8–13 Hz) and beta (14–30 Hz) bands at four electrodes: 12 (F3), 20 (C3), 50 (C4), and 60 (F4).

### Statistical Analysis

Mean EEG amplitude and PSD in the prefrontal and sensorimotor cortices were analyzed using three-way (type of contraction, side of body, side of brain) repeated-measure ANOVAs *via* SPSS (Version 25) at the two different time points (500–1,000 ms and 2,000–2,500 ms). When appropriate, simple contrasts between means were performed. Significance was set *a priori* at *p* < 0.05. All data are presented as mean ± standard error.

## Results

### EEG Amplitudes

**Figure 2:**
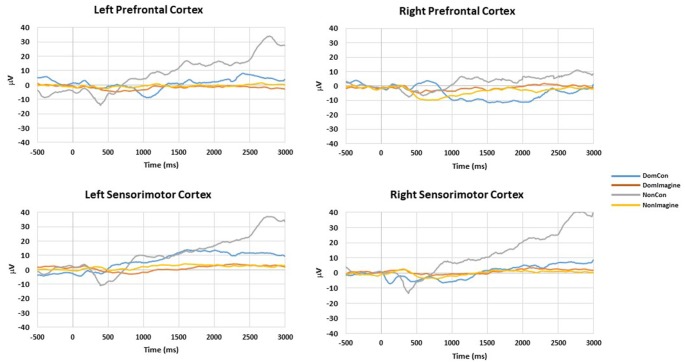
Amplitudes of right and left hemispheres of the prefrontal and sensorimotor cortices during DomCon, NonCon, DomImagine, and NonImagine 500 ms before to 3,000 ms after initiation of isometric elbow flexion.

EEG amplitudes for left and right hemispheres for DomCon, NonCon, DomImagine, and NonImagine in the sensorimotor and prefrontal cortices 500 ms before to 3,000 ms after initiation of tasks are shown in Figure 2. Similar pronounced amplitude differences are observed between the 500 and 1,000 ms and 2,000 and 2,500 ms range after initiation in both cortices.

### 500–1,000 ms After Initiation of Contraction

#### Prefrontal Cortex

**Figure 3:**
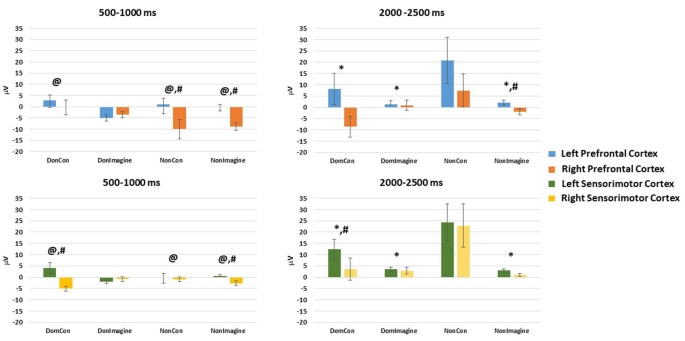
Mean amplitudes in the left and right hemispheres of the prefrontal and sensorimotor cortices 500–1,000 ms and 2,000–2,500 ms after initiation of isometric elbow flexion during DomCon, NonCon, DomImagine, and NonImagine. *Indicates difference from NonCon, ^@^indicates difference from DomImagine, ^#^indicates difference between left and right.

EEG amplitude in the prefrontal cortex 500–1,000 ms after initiation of contraction was not different between the type of contraction (physical vs. imagined; *F*_(1,14)_ = 2.186, *p* = 0.144) or side of the body (dominant vs. non-dominant; *F*_(1,14)_ = 1.321, *p* = 0.254). A difference did exist across sides of the brain (*F*_(1,14)_ = 13.125, *p* = 0.001). Furthermore, interactions did exist between the type of contraction and side of the brain (*F*_(1,14)_ = 3.482, *p* = 0.046) and between the side of the body and the side of the brain (*F*_(1,14)_ = 7.424, *p* = 0.008). Contrasts found EEG amplitudes in DomCon (*p* = 0.007), NonCon (*p* = 0.042), and NonImagine (*p* = 0.005) were different from DomImagine condition (Figure 3) in the prefrontal cortex 500–1,000 ms after contraction. Furthermore, differences between sides of the brain under this condition were observed in NonCon (*p* = 0.015) and NonImagine (*p* < 0.001) under these conditions.

#### Sensorimotor Cortex

EEG amplitude in the sensorimotor cortex 500–1,000 ms after initiation of contract was not different between the type of contraction (physical vs. imagined; *F*_(1,14)_ = 0.192, *p* = 0.662) or side of the body (*F*_(1,14)_ = 0.037, *p* = 0.847), but it was different across side of the brain (*F*_(1,14)_ = 7.735, *p* = 0.007). Furthermore, a two-way interaction between the type of contraction and side of the brain (*F*_(1,14)_ = 3.737, *p* = 0.049) as well as a three-way interaction (between type of contraction, side of the body, and side of the brain; *F*_(1,14)_ = 6.692, *p* = 0.012) were observed. Contrasts found that under these conditions, DomCon (*p* = 0.033) and NonImagine (*p* = 0.015) were different from DomImagine (Figure 3). Furthermore, differences between sides of the brain at this time point were observed in DomCon (*p* = 0.001) and NonImagine (*p* < 0.001).

Topographical plots of each condition 500–1,000 ms after contraction initiation display low levels of EEG activity during imagine contraction conditions compared to physical contraction conditions (Figure 3).

### 2,000–2,500 ms After Initiation of Contraction

#### Prefrontal Cortex

EEG amplitude in the prefrontal cortex 2,000–2,500 ms after initiation of the contraction was not different between the type of contraction (physical vs. imagined; *F*_(1,14)_ = 2.652, *p* = 0.110), side of the body (dominant vs. non-dominant; *F*_(1,14)_ = 2.281, *p* = 0.137), or between sides of the brain (*F*_(1,14)_ = 1.765, *p* = 0.190). However, a significant interaction between the type of contraction and side of the brain (*F*_(1,14)_ = 3.264, *p* = 0.043) as well as a significant three-way interaction (between type of contraction, side of the body, and side of the brain; *F*_(1,14)_ = 3.865, *p* = 0.0237) did exist. Contrasts revealed that EEG amplitude in the prefrontal cortex at this time point were lower in DomCon (*p* = 0.046), DomImagine (*p* = 0.035), and NonImagine (*p* = 0.042) compared to NonCon (Figure 3). Differences between sides of the brain under this condition were observed in DomCon (*p* = 0.042) and NonImagine (*p* = 0.004).

#### Sensorimotor Cortex

EEG amplitude in the sensorimotor cortex 2,000–2,500 ms after initiation of contract was different between the type of contraction (physical vs. imagined; *F*_(1,14)_ = 5.447, *p* = 0.023) and side of the body (dominant vs. non-dominant; *F*_(1,14)_ = 9.008, *p* = 0.004), but not between sides of the brain (*F*_(1,14)_ = 2.628, *p* = 0.110). A significant interaction between the type of contraction and side of the brain did exist (*F*_(1,14)_ = 7.468, *p* = 0.008). Contrasts revealed that DomCon (*p* = 0.018), DomImagine (*p* = 0.003), and Nonimagine (*p* = 0.004) displayed lower amplitudes than NonCon condition (Figure 3) under this condition. Furthermore, the left and right sides of the brain during DomCon in the sensorimotor cortex at this time point were different (*p* = 0.02).

**Figure 4:**
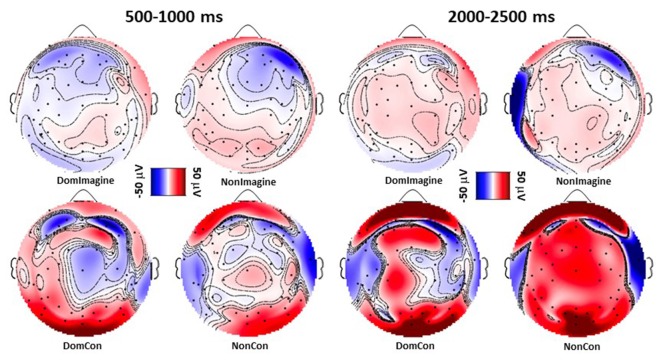
Topographical plots following sensory layout of EEG amplitude 500–1,000 ms and 2,000–2,500 ms after initiation of isometric elbow flexion during DomCon, NonCon, DomImagine, and NonImagine. Red and blue indicate positive and negative voltage amplitudes, respectively, while darker shades represent higher magnitudes.

Similar to the topographical plots at 500–1,000 ms, 2,000–2,500 ms after initiation imagine contraction conditions displayed lower levels of EEG activity than physical contractions (Figure 4). Furthermore, EEG activity appeared to increase from 500–1,000 ms to 2,000–2,500 ms during the physical contractions.

### Power Spectral Densities

**Figure 5:**
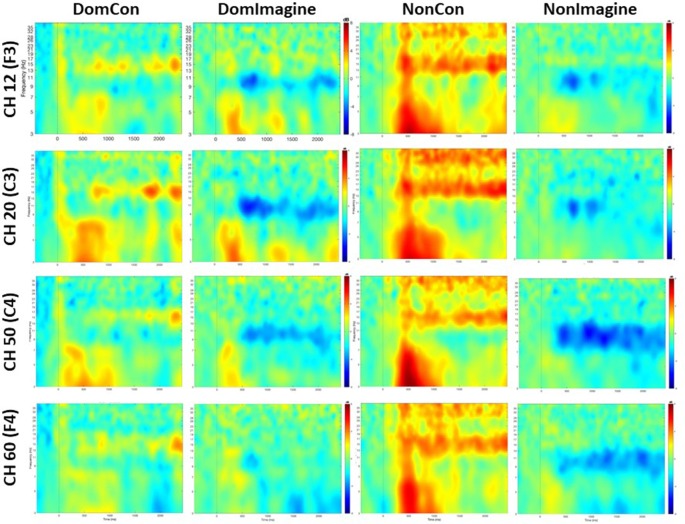
Time-frequency maps averaged across subjects for each channel and condition.

**Figure 6:**
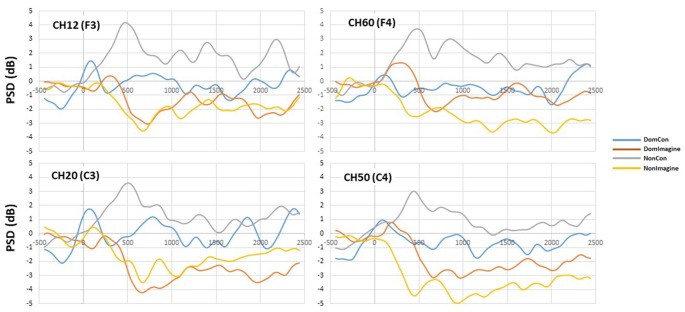
Power spectral density (PSD) curves in the alpha (8–13 Hz) band in channels 14(F3), 20 (C3), 50 (C4), and 60 (F4).

**Figure 7:**
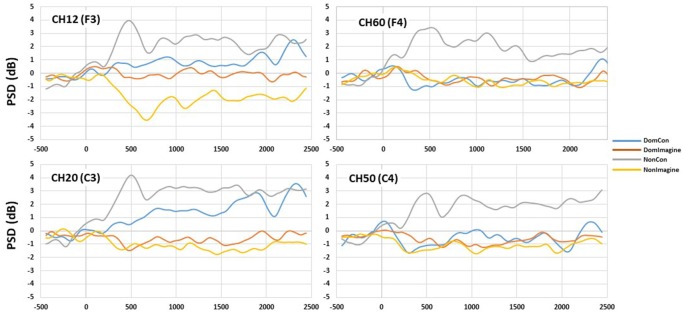
PSD curves in the beta (14–30 Hz) band in channels 14(F3), 20 (C3), 50 (C4), and 60 (F4).

**Figure 8:**
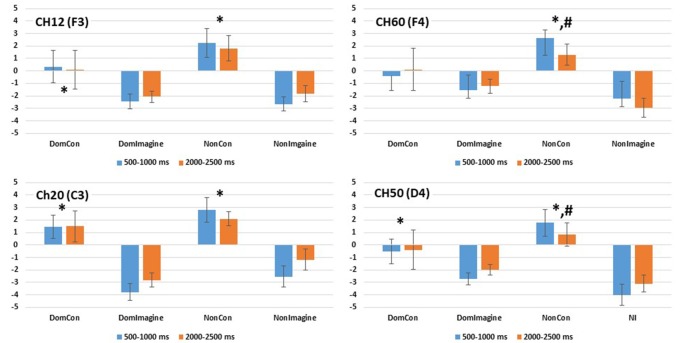
PSD in the alpha (8–13 Hz) band in channels 14(F3), 20 (C3), 50 (C4), and 60 (F4) 500–1,000 ms and 2,000–2,500 ms after initiation of contraction. *Indicates difference from imagined contraction. ^#^Indicates difference from contraction on dominant side of body.

**Figure 9:**
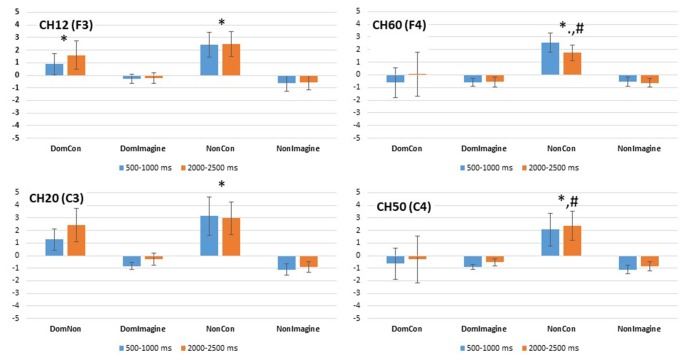
PSD in the beta (14–32 Hz) band in channels 14(F3), 20 (C3), 50 (C4), and 60 (F4) 500–1,000 ms and 2,000–2,500 ms after initiation of contraction. *Indicates difference from imagined contraction. ^#^Indicates difference from contraction on dominant side of body.

PSDs for channels 12 (F3), 20 (C3), 50 (C4), and 60 (F4) 500 ms before to 2,500 ms after initiation of tasks are shown in Figure 5. PSD curves representing ERSP data in the alpha and beta bands in the electrodes of interest are shown in Figures 6, 7. Mean PSD powers at 500–1,000 ms and 2,000–2,500 ms after initiation of contract in alpha and beta bands are shown in Figures 8, 9.

#### Prefrontal Cortex

PSD in the alpha band in channel 12 (F3) was different across type of contraction (*F*_(1,14)_ = 14.169, *p* = 0.002), but was not different across side of the body (*F*_(1,14)_ = 1.643, *p* = 0.221) or time (500–1,000 ms vs. 2,000–2,500 ms; *F*_(1,14)_ = 0.093, *p* = 0.764; Figure 8). Similar findings were observed in the beta band in channel 12 (F3) with a difference in PSD across the type of contraction (*F*_(1,14)_ = 5.557, *p* = 0.035), but not across side of the body (*F*_(1,14)_ = 0.483, *p* = 0.449) or time (*F*_(1,14)_ = 1.252, *p* = 0.283; Figure 9).

Similar findings in PSD in the alpha band were also observed in channel 60 (F4) with a difference across type of contraction (*F*_(1,14)_ = 13.149, *p* = 0.003), but not side of the body (*F*_(1,14)_ = 1.643, *p* = 0.221) or time (500–1,000 ms vs. 2,000–2,500 ms; *F*_(1,14)_ = 0.093, *p* = 0.764; Figure 8). Interestingly, an interaction between side of the body and time (*F*_(1,14)_ = 6.846, *p* = 0.020) was present in the alpha band of channel 60 (F4). In the beta band of channel 14 (F3), there was a difference in PSD across the type of contraction (*F*_(1,14)_ = 12.638, *p* = 0.003), but not across side of the body (*F*_(1,14)_ = 2.103, *p* = 0.169) or time (*F*_(1,14)_ = 0.001, *p* = 0.974; Figure 9).

#### Sensorimotor Cortex

PSD in the alpha band in channel 20 (C3) was different between type of contraction (*F*_(1,14)_ = 34.645, *p* < 0.001), but was not different across side of the body (*F*_(1,14)_ = 2.419, *p* = 0.146) or time (500–1,000 ms vs. 2,000–2,500 ms; *F*_(1,14)_ = 1.102, *p* = 0.314). Similar findings were observed in the beta band in channel 20 (C3) with a difference in PSD across the type of contraction (*F*_(1,14)_ = 8.515, *p* = 0.012), but not across side of the body (*F*_(1,14)_ = 0.447, *p* = 0.516) or time (*F*_(1,14)_ = 1.911, *p* = 0.190).

Similar findings for PSD in the alpha band were also observed in channel 50 (C4) with a difference across type of contraction (*F*_(1,14)_ = 15.684, *p* = 0.001), but not across side of the body (*F*_(1,14)_ = 0.253, *p* = 0.623) or time (500–1,000 ms vs. 2,000–2,500 ms; *F*_(1,14)_ = 0.167, *p* = 0.689; Figure 8). Interestingly, interactions between type of contraction and side of the body (*F*_(1,14)_ = 19.362, *p* = 0.001) as well as side of the body and time (*F*_(1,14)_ = 15.502, *p* = 0.001) were present in the alpha band of channel 50 (C4). In the beta band in channel 50 (C4), there were no differences in ERSP across the type of contraction (*F*_(1,14)_ = 2.676, *p* = 0.367), side of the body (*F*_(1,14)_ = 2.315, *p* = 0.150), or time (*F*_(1,14)_ = 0.869, *p* = 0.974; Figure 9).

## Discussion

This study is the first to present primary findings regarding differences in MRCP and ERSP during non-dominant and dominant physical and imagined unilateral tasks. Physical tasks produced bilateral increases in MRCP in both the sensorimotor and prefrontal cortices. Physical contractions appeared to generate more brain activity than imagined contractions overall. Physical contractions generally elicited positive changes in alpha and beta band activity in both the sensorimotor and prefrontal cortices. On the other hand, imagined contractions displayed negative changes in alpha and beta band activity in both cortices. Greater beta band activity was observed only during physical contractions. Nondominant physical contractions also elicited the greatest MRCP and frequency band changes out of all conditions and these changes were bilateral in nature. Instances of laterality appeared to only be observed during DomCon. When looking at approximately the 500-ms mark in both MRCP and frequency bands across all tasks, a phenomenon can be observed in which both measurements display obvious peak projections. For example, a negative reflection during NonDom MRCP was accompanied by a large increase in ERSP.

The general findings of this study were consistent with those of previous research. MRCP changes during physical contractions mimicked the pattern observed by Gilden et al. (1966) of an increasing positive deflection representative of sustained maximal muscle contraction. Additionally, activation of both the sensorimotor and prefrontal cortices during physical and imagined contractions support the observations documented by other studies measuring activity in similar brain regions before and after a physical or mental training intervention (Yue and Cole, 1992; Siemionow et al., 2000; Fang et al., 2001). Changes in ERD within beta bands during the mental contractions in both cortices were consistent with those found with a mental hand grasping task (Bian et al., 2018). Likewise, the decreased PSD in the alpha band observed here during imagined contractions is similar to those reported by Chen and associates during a mental hand rotation task (Chen et al., 2013). The increased PSD observed in the beta band during physical contractions is also consistent with the findings observed by others (Neuper and Pfurtscheller, 2001; Wang et al., 2015). The observation of greater changes exhibited by NonCon is similar to those reported by Yuan et al. (2010). Unlike previous studies, a relationship between MRCP and frequency band activities was observed. At approximately 500 ms after stimulus, MRCP and both alpha and beta bands can be seen to produce pronounced deflections, suggesting that the sustained portion of the task has been reached.

Out of all the testing conditions, non-dominant physical contractions elicited the greatest changes in MRCP and frequency bands, and these changes were bilateral in nature. This suggests that the brain does not fully operate with laterality as is conventionally believed, especially during contractions in the non-dominant limb. This could be due to less familiarity and reduced motor control in the non-dominant side, requiring more neural input and attentional resources to execute the task. Handedness may influence these findings. For example, left-handed or ambidextrous individuals may display varying amounts of brain activity in different regions that are associated with their adaptation to a right-handed world (in the case of left-handed) or development of robust motor schemas (ambidextrous). There are no studies evaluating brain activity and detailing participants’ limb dominance while performing tasks with each limb. Farthing et al. (2005) did find strength and muscle increases *via* electromyography to be more prominent on the non-dominant side when training the dominant side for a cross-education investigation. Strength training the dominant arm resulted in the immobilized non-dominant arm to experience no decrease in muscle size while no training resulted in a 15% decrease in size (Farthing, 2009). The dominant arm tended to have greater strength and motor control over the non-dominant arm, but training the non-dominant arm had greater potential for change on the respective side than training of the dominant side (Farthing et al., 2005). Despite this finding, training the dominant arm continues to be the more popular approach, especially in investigations examining the value and mechanisms underlying cross-education. In a clinical setting, the luxury of predetermining which limb may be compromised does not exist. As a result, research should be more inclusive towards measuring the effect and responses of both dominant and non-dominant sides. The importance of studying the effects of training may provide a greater understanding of the role bilateral activity plays in the cross-education phenomenon and how to effectively use this strategy for rehabilitation purposes.

Understanding changes in brain activity during sustained tasks on non-dominant and dominant sides have great implications for rehabilitation and treatment in clinical populations. For instance, mental isometric imagery tasks are a widely used technique in many therapy programs, particularly in stroke patients with unilateral limb weakness and compromised motor function (Liu et al., 2014). Additionally, further mapping and understanding of the specific responses dominant and non-dominant tasks elicit in both MRCP and band frequencies may shed light on the neural mechanism responsible for cross-education, which may be valuable for treating patients suffering from a one-sided injury or immobilization. Current literature suggests that a high-order neural mechanism is responsible for the crossover of strength and mobility and preservation of muscle size from one trained limb to the opposite homologous untrained limb (Cirer-Sastre et al., 2017). The present study showed bilateral activity in brain hemispheres within the sensorimotor and prefrontal cortices during unilateral tasks, suggesting that brain activity is developing a motor schema using one side of the body that could be used by the opposite side. This, in turn, could have a part in the transfer effect commonly seen with this unique type of training. Brain activity may also be used as a diagnostic measurement for assessing those who have suffered from stroke, hemiparalysis, immobilization, etc., in order to develop specific treatments. Lastly, identifying brain activity trajectories throughout mental tasks may be worthwhile when developing brain–computer interfaces for advanced prosthetics and therapies. As more advanced multi-joint prosthetics are developed, sustained precise neural input is required to drive the equipment for task execution (Brauchle et al., 2015).

### Limitations

The results of this investigation are limited by the methods and approaches to data analyses used. FMRI investigations would likely provide new information regarding which brain areas were active during sustained physical and imagined isometric contraction. Different methodological and analytical approaches may have provided unique insight into the brain responses to physical movement and future research should explore these techniques.

Additionally, all participants within the study were right-handed. Left-handed individuals might require fewer neural and attentional resources to perform the same tasks as they might be more accustomed to utilizing their right hand in a predominantly right-handed society. On the other hand, ambidextrous individuals may display equal amounts of brain activation regardless of which hand is used.

Another limitation of this study was requiring participants to perform only five trails for each task. Only five were used in this investigation because it has been shown that contractile function of the muscle begins to decline after five isometric repetitions (Ali et al., 2014). Additionally, participants were asked to perform many testing conditions, increasing the likelihood of muscle fatigue. However, investigating the effects of prolonged activity and muscle fatigue as a consequence of performing repetitive tasks on brain activity may be valuable for physical therapy treatment. Similarly, the order of conditions was not randomized in this study. It has previously been demonstrated that motor priming can occur due to acute mental or physical practice of a task or similar task prior to therapeutic interventions (Stoykov and Madhavan, 2015). However, this study demonstrated elevated MRCPs in the physical contractions compared to the mental contractions. If motor priming was present, it would be expected that later trials, in this case, the imagined contractions, would experience elevated MRCPs. Further, much of the current literature suggests the use of motor priming as a therapeutic enhancement to physical therapy. There is, therefore, a lack of research indicating the expected effects of physical contractions on the MRCPs during subsequent mental contractions.

## Conclusion

In conclusion, the present study showed that physical and mental unilateral isometric elbow flexion generates bilateral brain activity, with physical contractions showing greater sustained changes. Physical contractions also displayed positive changes in alpha and beta bands in the sensorimotor and prefrontal cortices, while imagined contractions showed negative changes in alpha and beta band activity in both cortices. Both MRCP and frequency bands across all tasks elicited a phenomenon at approximately the same time in which both measurements display extreme peak projections. Although unilateral physical and mental isometric contractions in non-dominant and dominant limbs facilitated observable changes in brain activity, the lack of similarity between physical and imagined contractions suggests that imagined contractions may not be the most effective surrogate for physical contractions in a clinical setting. Future studies should continue examining non-dominant and dominant tasks, along with dynamic contractions, as it may prove valuable for understanding the neural changes and adaptations during mental imagery of physical movement along with practical clinical applications.

## Data Availability Statement

The datasets generated for this study are available on request to the corresponding author.

## Ethics Statement

The studies involving human participants were reviewed and approved by Elon University Institutional Review Board for the Protection of Human Subjects in Research. The patients/participants provided their written informed consent to participate in this study.

## Author Contributions

JM designed and presented the research idea and recruited participants and collected/processed data. MW designed the research idea. SB provided equipment, designed processing framework, and analyzed the data. SF designed and implemented the process for frequency analysis of data. MW and SB supervised the project. JM took lead in writing the manuscript with contributions from MW and SB. SF provided input regarding the interpretation and discussion of the frequency data.

## Conflict of Interest

The authors declare that the research was conducted in the absence of any commercial or financial relationships that could be construed as a potential conflict of interest.
