# Supplementary material for: Brain Activity During Unilateral Physical and Imagined Isometric Contractions
Source: Front Hum Neurosci. 2019 Nov 26;13:413. doi: 10.3389/fnhum.2019.00413 (PMC7004234; doi:10.3389/fnhum.2019.00413)
Supplement: Supplementary file 1 [file Data_Sheet_1.PDF]

Table 1. Descriptive statisitcs for participants

Sex	6 male; 9 female
Age	18.8 ± 1
Height	170.5 ± 11 cm
Weight	71.2 ± 13.1 kg
Left Grip strength	29.6 ± 8.7 kg
Right Grip strength	32 ± 9.6 kg

Values are means ± standard deviations.
