# Supplementary material for: Brain Activity During Unilateral Physical and Imagined Isometric Contractions
Source: Front Hum Neurosci. 2019 Nov 26;13:413. doi: 10.3389/fnhum.2019.00413 (PMC7004234; doi:10.3389/fnhum.2019.00413)
Supplement: Supplementary file 2 [file Data_Sheet_2.PDF]

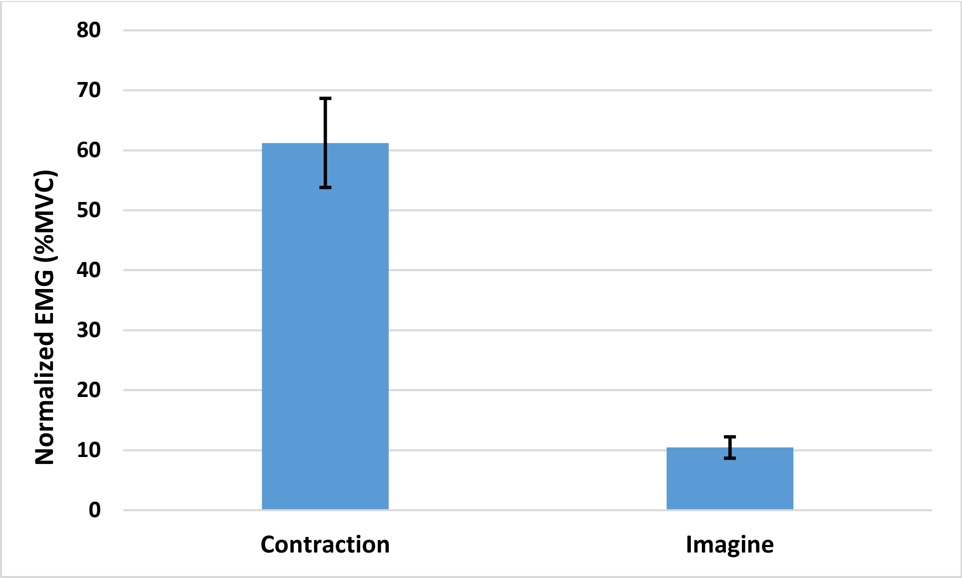

The data presented illustrates the EMG activity during physical contraction (average peak value during 5 bursts) and the peak value observed during imagined contractions. All data were normalized to the Maximum voluntary Isometric Contraction (MVC) of the same limb. MVC contractions were completed prior to the start of the experiment. An amplitude analysis was conducted by using a windowed RMS filter (window size = 0.125 seconds, window overlap = 0.0625 seconds) then dividing by the peak value during the MVC trial (EMGWorks Analysis version 4.7.3.0, Delsys, Inc., Boston, MA).
